# The Relationship between Personality Type and Acceptable Noise Levels: A Pilot Study

**DOI:** 10.1155/2013/902532

**Published:** 2013-11-14

**Authors:** Cliff Franklin, Laura V. Johnson, Letitia White, Clay Franklin, Laura Smith-Olinde

**Affiliations:** ^1^University of Arkansas at Little Rock, Audiology and Speech Pathology UP600, 2801 S. University Avenue, Little Rock, AR 72204, USA; ^2^University of Arkansas for Medical Sciences, Audiology and Speech Pathology, 4301 W. Markham Street, No. 702, Little Rock, AR 72205, USA; ^3^Missouri State University, Communication Sciences and Disorders, Professional Building, 901 S. National Avenue, Springfield, MO 65897, USA; ^4^University of Arkansas for Medical Sciences, Office of Educational Development, 4301 West Markham Street, No. 595 Little Rock, AR 72205, USA

## Abstract

*Objectives*. This study examined the relationship between acceptable noise level (ANL) and personality. ANL is the difference between a person's most comfortable level for speech and the loudest level of background noise they are willing to accept while listening to speech. *Design*. Forty young adults with normal hearing participated. ANLs were measured and two personality tests (Big Five Inventory, Myers-Briggs Type Indicator) were administered. *Results*. The analysis revealed a correlation between ANL and the openness and conscientious personality dimensions from the Big Five Inventory; no correlation emerged between ANL and the Myers-Briggs personality types. *Conclusions*. Lower ANLs are correlated with full-time hearing aid use and the openness personality dimension; higher ANLs are correlated with part-time or hearing aid nonuse and the conscientious personality dimension. Current data suggest that those more open to new experiences may accept more noise and possibly be good hearing aid candidates, while those more conscientious may accept less noise and reject hearing aids, based on their unwillingness to accept background noise. Knowing something about a person's personality type may help audiologists determine if their patients will likely be good candidates for hearing aids.

## 1. Background

Many factors may influence a person's success with hearing aids, for example, sound processing by the aid and the presence of background noise. However, the factors that underlie the hearing aid wearer's perception of success and benefit are not fully understood. For example, Hutchinson et al. [1] reported that two people with the same type and degree of hearing loss and no prior experience with hearing aids can perceive hearing aid benefit differently. To clinically assess patients' perceptions of hearing aid benefit, the subjective *Client Oriented Scale of Improvement* (COSI [2]) and the *Abbreviated Profile of Hearing Aid Benefit* (APHAB [3]) have been available for over 15 years and are widely used by audiologists.

Other research has focused on clinical variables that can be manipulated such as counseling, the fitting process, type of hearing aid, compression circuitry, and other hearing instrument adjustments. While these modifications can help give wearers more objectively measureable benefit with hearing aids, there are still some who feel that they never achieve success with hearing aids [4–6]. In light of such research findings, it seems reasonable to think there may be as yet unidentified wearer-related variables that affect a person's perceived benefit/success with hearing aids. One such variable that may yield part of the answer with regard to perceived hearing aid benefit is the psychological variable of “personality” [1, 5, 7, 8].

Relatively little research has been done on personality and audiology and particularly with regard to perceived hearing aid benefit. Cox et al. [5] reported significant relationships in seniors who wear hearing aids between the APHAB and the extroversion-introversion dimension of the Myers-Briggs Type Indicator and also a significant relationship between the APHAB and the State-Trait Anxiety Inventory. Subjects perceiving greater hearing aid benefit scored stronger in the extroversion category, and those perceiving less benefit from hearing aids scored higher in the anxiety category [1, 3, 5].

One well-known factor underlying hearing aid rejection is the presence of background noise. Almost half (49%) of those individuals who have rejected and returned their hearing aids cited difficulty hearing in noise as a reason for the return [9]. Hearing aids are improving in their ability to ease listening in background noise; however, many people have difficulty following conversations in its presence [10–12]. To investigate the tolerance of background noise while listening to speech, Nabelek et al. [10] created the Acceptable Noise Level (ANL) technique. This test measures the loudest level of background noise that a person will accept and report speech listening is not compromised.

### 1.1. Acceptable Noise Level

ANL is the difference between a person's most comfortable listening level for speech and the loudest level of background noise that a person is willing to accept without becoming tense or tired while listening to speech. ANL is measured by first finding a person's most comfortable level (MCL) for speech. Background noise is then added and increased until the person indicates the noise is at the highest tolerable level without becoming tense or tired. The most intense acceptable background noise level (BNL) is then subtracted from MCL to yield the ANL (ANL = MCL − BNL [10]). Therefore, smaller ANLs indicate that listeners will accept higher levels of background noise while listening to speech and larger ANLs the reverse.

Many variables have been demonstrated to have little to no effect on ANL, including gender [13], age [10, 14], hearing loss [10, 12], the language of the stimulus [15], and use of hearing aids [16, 17]. However, Mueller et al. [17] reported that activating digital noise reduction (DNR) in a hearing aid will significantly lower the ANL versus without DNR and also without hearing aids. Wu and Stangl [18] found a similar effect on ANL when DNR was employed.

ANL has been shown to be a more accurate measure in predicting hearing aid success than objective measures such as speech understanding in noise or degree of hearing loss, reaching an accuracy of 85% in Nabelek et al. [12]. These researchers demonstrated that, the smaller the ANL, the more likely the individual wears hearing aids full-time, and the larger the ANL, the more likely the individual is wearing hearing aids part-time or not at all. Freyaldenhoven et al. [19] reported a correlation between the ANL and two subscales of the APHAB (Ease of Communication and Background Noise), which predicted hearing aid success with 91% accuracy.

### 1.2. Personality

Personality has been shown to affect areas of life such as coping with stress, dealing with crises, and job performance [8, 20, 21]. It is reasonable, therefore, to think that personality may affect other areas of life such as coping with disability and hearing loss, adapting to new situations, and tolerance of background noise. Geen [22] reported that extroverts chose higher levels of intensity than introverts for completing a paired-associates learning task, suggesting that personality type can play a role in preferred auditory levels. Perhaps knowledge of personality and how it relates to ANL can help determine why some people have large ANLs and some have small ANLs.

Many personality tests have been used to assess individuals' personality. One common test is the Big Five Personality Test. The specific personality areas covered by the “Big Five” are openness to new experiences, conscientious, extraverted, agreeable, and neurotic/high strung, with their respective opposites being closed-minded, disorganized, introverted, disagreeable, and calm/relaxed [20, 23, 24]. Goldberg [23] studied the repeatability and variance of the five-factor model and found that the Big Five factors were replicable between samples and that no additional factors beyond the Big Five, for example, religiosity, were replicable. Other research has shown that the five-factor model is a useful tool for assessing personality and individual differences. For example, people who are open to new experiences tend to be imaginative, artistic, and cultured, while people who are neurotic or high strung tend to be anxious, worried, emotional, and insecure [20].

Another common personality test is the Myers-Briggs Type Indicator (MBTI). Myers and Briggs added a fourth dimension, judging-perceiving (J-P), to Jung's [25] three personality dimensions: extroversion-introversion (E-I), sensing-intuition (S-N), and thinking-feeling (T-F). Jung contended that individuals had a dominant and nondominant aspect to these dimensions. For example, an extroverted person tends to be focused on the world around them, and an introverted person tends to be self-reflective. The MBTI assigns the appropriate letter to indicate the dominant aspect, for example, E for extroversion, for each of the four dimensions, resulting in 16 possible personality types. The MBTI has been used in several research studies for assessing personality and its relation to many areas in a person's life, including perceived benefit from and/or use of hearing aids [1, 7, 8, 26]. Also, it has been shown that the MBTI may be a useful tool in aural rehabilitation and counseling. In addition, Nichols and Gordon-Hickey [27] found a relationship between the personality attribute of self-control and ANL. Listeners with higher levels of self-control had smaller ANLs than those with lower levels of control. If a person's personality is known, the hearing healthcare professional can target aural rehabilitation specifically to fit the individuals needs and how they deal with situations [7].

Since the ANL measure is a predictor as to which individuals will wear hearing aids on a full-time basis, it has been suggested that the ANL test can be used as a counseling tool [28]. ANL can give audiologists insight as to which individuals need more counseling regarding realistic expectations and which ones need more advanced technology such as directional microphones or assistive listening devices to help ease listening in the presence of background noise. Combining the indications of the ANL test and knowing a little about an individual's personality may give audiologists additional information that informs counseling of their patients to achieve better success with hearing aids. 

 The purpose of this pilot study was to determine if certain personality types or specific dimensions of personality are associated with an individual's ANL. 

## 2. Methods

### 2.1. Participants

Forty young adults (age range = 18–30 years) participated in this study. Recruited from Missouri State University, all participants had normal hearing (≤25 dB HL thresholds at octaves from 250 to 8000 Hz in soundfield) and normal middle ear function. Additional inclusion criteria were English as their first language and no cognitive or neurological deficits. 

### 2.2. Stimuli and Materials

Otoscopy was conducted, and middle ear function was tested using 226 Hz tympanometry with a GSI Tympstar. Jerger type A tympanograms were required for each participant to continue in the study. Pure-tone audiometry was performed using a Madsen Astera audiometer. The audiometer was calibrated according to the American National Standards Institute (ANSI S3.6-1996) standards, and the sound treated booth met the requirements for ambient noise levels (ANSI S3.1-1991). Each participant sat three feet in front of a loudspeaker at 0° azimuth. Soundfield thresholds were determined for each participant at 0.25, 0.5, 1, 2, 4, and 8 kHz.

Acceptable noise levels (ANL) were determined using the Arizona Travelogue (Cosmos, Inc.) as the primary stimulus and the R-SPIN [29] multitalker speech babble as the background noise. Both the running speech and speech babble were presented through a single speaker from a compact disc player routed through the audiometer. The most comfortable level (MCL) for the primary stimulus and the loudest acceptable background noise level (BNL) for the speech babble were established for each participant. Acceptable noise levels were determined by subtracting the BNL from the MCL [10].

Each participant also completed the Big Five Personality Test and an abbreviated version of the Myers-Briggs Type Indicator to assess their personality. The Big Five Personality Test, also known as the Big Five Inventory (BFI), assesses five personality dimensions that are sometimes referred to as the OCEAN model of personality (open to new experiences, conscientious, extraverted, agreeable, and neurotic/high strung). The inverses of these categories are closed-minded, disorganized, introverted, disagreeable, and calm/relaxed. The BFI contains 44 items which contain a short phrase, for example, “Has an assertive personality” and “Is sometimes shy, inhibited.” The participants rate themselves for each item on a 1 (disagree strongly) to 5 (agree strongly) Likert scale. Each dimension receives a rating from 0% to 100%. A higher percent means the person's personality is more like that particular dimension category, and a lower percent means they are more like the inverse of that particular dimension [20, 23, 24].

The abbreviated version of the Myers-Briggs Type Indicator (MBTI) contains 72 items that assess four personality dimensions: extroversion/introversion (E-I), sensing/intuition (S-N), thinking/feeling (T-F), and judging/perceiving (J-P). The respondent marks “yes” or “no” to a series of statements, for example, “You enjoy having a wide circle of acquaintances,” and is assigned one aspect per dimension along with a measure of strength. The strength measure ranges from 1 to 100, one being very weak and 100 being very strong. For example, one of the personality types could be ESTJ, signifying that the person scored higher on the questions related to extroversion, sensing, thinking, and judging [1, 30, 31]. The BFI and the MBTI were administered and scored on an online website.

### 2.3. Procedures

For each participant, otoscopy and tympanometry were first conducted to confirm normal middle ear function, and then pure-tone audiometry was carried out to ensure soundfield thresholds ≤25 dB HL. The most comfortable level (MCL) and background noise level (BNL) measures were obtained next. Instructions for the MCL and BNL measures (the appendix) were typed, and each participant could read the instructions while the researcher read them aloud. Any questions were answered prior to testing [12].

Acceptable noise levels were determined for each participant by finding their individual MCL measures for recorded speech and their acceptable BNL. The BNL was subtracted from the MCL to find their ANL. The participants were told that the goal was to determine the loudest level of background noise that they can accept without becoming tense or tired while listening to the running speech. The participant listened to the primary stimulus and running speech and indicated an increase (thumbs-up) or decrease (thumbs-down) in level. The researcher controlled the stimulus level through the audiometer. Participants indicated their MCL had been reached by raising their hand in the air. Initially, the stimulus was presented in 5 dB steps then was presented in 2 dB steps. Once the MCL was established, the running speech continued at the level indicated by the participant. Next, the speech babble background noise was presented, and the same increase/decrease technique was used to determine the participant's loudest acceptable background noise level [10]. 

After ANL testing, each participant completed the Big Five Inventory (BFI) and the abbreviated Myers-Briggs Type Indicator (MBTI) online with scoring also completed online. The percent rating for each of the five dimensions of personality (BFI) and the personality types (MBTI) comprised the scores. The participants were classified as one of the 16 possible types in the MBTI, and the strength of each personality dimension was recorded. 

### 2.4. Statistical Analysis

 Descriptive statistics were calculated for the MCL, BNL, BFI, and MBTI. Regression analyses were run between each of the Big Five Inventory dimensions and ANL as well as each pair of personality traits in the Myers-Briggs test and the ANL.

## 3. Results

 The values reported were averaged across all 40 participants. The data on MCL, BNL, and ANL are listed in Table 1. The mean group MCL was 41.7 dB HL (SD = 7.6 dB) with a mean BNL of 34.7 dB HL (SD = 9.1 dB) and a mean group ANL of 7.0 dB (SD = 5.0 dB). Group mean percentages for the nine rated dimensions of personality type on the two instruments used, correlations between those dimensions and ANL, and significance values for each regression can be seen in Table 2. Group mean percentages on the BFI ranged from a high of 74.9 on “conscientious” to a low of 27.3 for “openness.” Recall that the BFI scale is from 0% to 100% with a higher value indicative of possessing “more” of that dimension. The Myers-Briggs personality dimensions were given a score from −100 to 100. Extroversion, sensing, thinking, and judging were given positive values (1 to 100), and introversion, intuition, feeling, and perceiving were given negative values (−1 to −100). The farther away the value is from zero, the stronger the participants scored in that category. The data obtained in the study indicate that the 40 participants are, as a group, slightly extroverted (8.9), slightly intuitive (−7.4), mildly feeling (−20.9), and most strongly judging (46.4).

**Figure 1:**
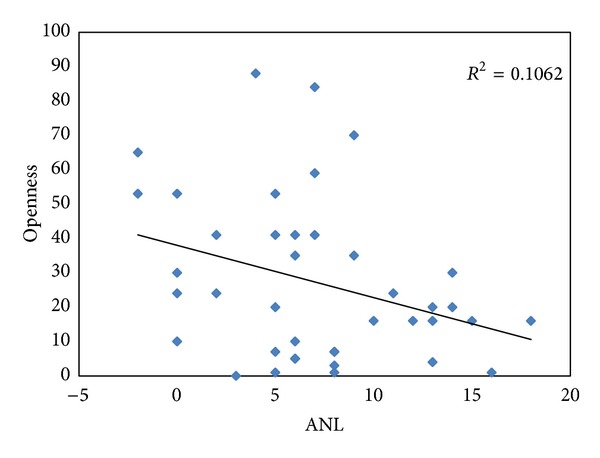
Regression analysis showing the relationship between ANL and the openness category from the Big Five Inventory.

**Figure 2:**
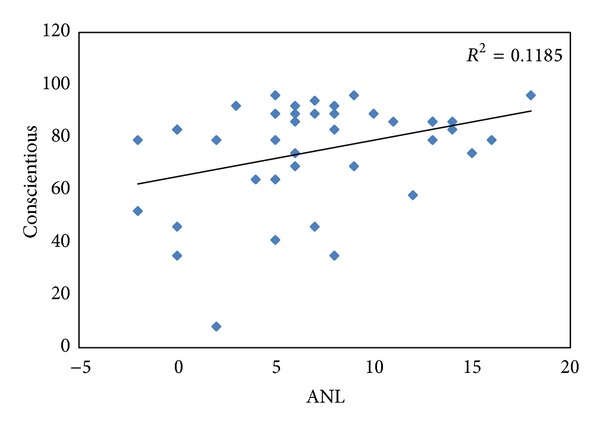
Regression analysis showing the relationship between ANL and the conscientious category of the Big Five Inventory.

Pearson product correlations were used to determine whether a relationship existed between any of the personality dimensions and ANL. These data are listed in Table 2. A weak, but statistically significant, inverse correlation (Figure 1) was found between the ANL value and the BFI category of openness (*r* = 0.326, *P* = 0.040), with *r* ^2^ = 0.106. The inverse correlation indicates that, as the level of openness decreases, the magnitude of the ANL increases. Another weak, but statistically significant, direct correlation (Figure 2) was indicated between the ANL value and the BFI category of conscientious (*r* = 0.208, *P* = 0.030), with *r* ^2^ = 0.119. In contrast to the openness dimension, as the conscientious dimension strength increases, so too does the magnitude of the ANL. None of the remaining correlations reached statistical significance.

## 4. Discussion

The purpose of this pilot study was to determine if certain personality types or specific dimensions of personality contribute to acceptable noise levels. Each participant's five personality dimensions from the Big Five Inventory and their personality type from the Myers-Briggs Type Indicator were compared to their individual ANL score. Regression analysis including a Pearson product correlation was used to determine if there is a relationship between any of the personality types and ANL. 

It was found that the openness and conscientious personality dimensions from the Big Five Inventory have a small effect on acceptable noise levels. The participants who were more open to new experiences had lower ANLs, suggesting they were more willing to accept higher levels of background noise. The participants who were more conscientious had larger ANLs, suggesting they were less willing to accept the presence of background noise. 

People who are open to new experiences tend to be tolerant, imaginative, artistic, and cultured [20]. Therefore, people who scored higher on the openness personality dimension probably tolerated the annoyance of higher background noise resulting in lower ANLs. The openness personality type describes about 11% of the variance in ANL scores (*r* ^2^ = 0.106). People who are conscientious tend to be thorough, meticulous, organized, and responsible. They attend to things with more focus and place more importance on attending to a task. They also desire fewer distractions when focusing on a task [20]. Therefore, people who scored higher on the conscientious personality dimension probably placed more importance on listening to the target stimuli consequently rejecting more background noise. The conscientious personality type describes about 12% of the variance in ANL scores (*r* ^2^ = 0.119). No significant correlations were found between the other personality dimensions (extraverted, agreeable, and neurotic/high strung) from the Big Five Inventory.

Alworth et al. [26] found a positive relationship between ANL and the sensing and judgment personality dimensions of the Myers-Briggs Type Indicator (larger ANL and higher score for both sensing and judging). Hutchinson et al. [1] found a positive relationship between perceived benefit of hearing aids and the MBTI thinking personality dimension (greater perceived benefit and higher score for thinking). Hutchinson et al. [1] also found a negative relationship between perceived hearing aid benefit and the intuition and perception personality dimensions of the Myers-Briggs Type Indicator. This finding indicates that the participants who perceived little benefit from their hearing aids had higher scores for intuition and perceiving. In the present study, none of the four dimensions rated in the MBTI were found to correlate with ANL. Possible reasons for this finding include the small sample size of 40 participants in the current study and the large number of possible personality types on the MBTI.

Results of the current study support the hypothesis that at least some dimensions of personality type, as measured by the Big Five Inventory, are related to the acceptance of background noise. 

## 5. Conclusion

 If the relationships between personality type and ANL hold true for listeners with hearing loss, then knowing something about a person's personality type may help audiologists determine if that client will most likely be a good candidate for hearing aids. Since low ANLs are correlated with full-time use of hearing aids and the openness personality dimension, it can be concluded that those who are more open to new experiences may have a predisposition to accepting more noise and possibly be better hearing aid candidates. Because high ANLs are correlated with part-time or nonuse of hearing aids and the conscientious personality dimension, we might conclude that those who are more conscientious may have a predisposition to accepting less noise and possibly reject hearing aids based on their lack of willingness to accept background noise.

 Future research should examine the relationship between personality type and acceptance of background noise in impaired hearing listeners. Also, the use of the Keirsey Temperament Sorter to determine individual Myers-Briggs personality types may provide additional significant information instead of using the online abbreviated Myers-Briggs Type Indicator used in the present study. Future research should also focus on incorporating the Big Five Inventory into other areas of audiology such as aural rehabilitation and creating an abbreviated measure of personality that would be more time-sensitive to use in a clinical setting.

## Figures and Tables

**Table 1:** Most comfortable levels (MCL) for speech in dB HL, background noise levels (BNL) in dB HL, and acceptable noise levels (ANL) in dB.

	Mean	Standard deviation	Range
MCL	41.7	7.6	30–63
BNL	34.7	9.1	22–63
Resulting ANL	7.0	5.0	−2–18

**Table 2:** Means for personality types and regression statistics for ANL and personality types/categories.

Category	Mean	*r*	*r* ^2^	Significance
Openness	27.3	0.326	0.106	0.040*
Conscientious	74.9	0.344	0.119	0.030*
Extraverted	55.4	0.208	0.043	0.198
Agreeable	68.4	0.194	0.038	0.231
Neurotic/high strung	37.5	0.128	0.016	0.431
Extraverted/introverted	8.9	0.197	0.039	0.224
Sensing/intuition	−7.4	0.038	0.001	0.817
Thinking/feeling	−20.9	0.127	0.016	0.433
Judging/perceiving	46.4	0.239	0.057	0.137

*Significant at the 0.05 level.

## Appendix

*Instructions for Establishing MCL*. You will listen to a story through a loud speaker. After a few seconds, select the loudness of the story that is most comfortable for you, as if listening to a radio. You will make adjustments to the volume by giving a thumbs-up or thumbs-down, indicating which direction you want the volume to go. First, turn the loudness up until it is too loud and then down until it is too soft. Finally, select the loudness level that is the most comfortable for you.

*Instructions for Establishing BNL*. Next, you will listen to the same story with the background noise of several people talking at the same time. After you have listened to this for a few seconds, select the level of background noise that is the most you would be willing to accept or “put up with” without becoming tense or tired while following the story. First, turn the noise up until it is too loud and then down until the story becomes very clear. Finally, adjust the noise (up and down) to the maximum noise level that you would be willing to “put up with” for a long time while following the story.
